# LLMs build inconsistent knowledge graphs from one immune-checkpoint corpus: a five-axis benchmark and consensus precision–coverage protocol

**DOI:** 10.3389/fimmu.2026.1937268

**Published:** 2026-08-18

**Authors:** Qiang Xie, Yang Fang, Yanan Hu

**Affiliations:** 1 Department of Surgical Oncology, The First Affiliated Hospital of Bengbu Medical University, Bengbu, Anhui, China; 2 Department of Laboratory Medicine, The Third Affiliated Hospital of Zhengzhou University, Zhengzhou, Henan, China; 3 Department of Infectious Diseases, The First Affiliated Hospital of Bengbu Medical University, Bengbu, Anhui, China

**Keywords:** biomedical knowledge graphs, immune checkpoint inhibitors, knowledge graph embedding, large language models, LLM-as-a-judge, model consensus, text mining

## Abstract

Large language models (LLMs) are increasingly used to mine biomedical literature into knowledge graphs (KGs), yet whether independent models agree when given the same text is largely untested. We passed 9,978 immune-checkpoint PubMed abstracts through four LLMs (DeepSeek, GLM, Kimi, and Qwen) under an identical prompt and an eight-entity-type schema. This yielded 140,462 triples and a 95,981-edge union graph. We benchmarked the four graphs along five axes: extraction, correctness against a human expert, topology, embedding-based link prediction, and external validation. Extraction volume varied more than twofold (20,122–45,957 triples), and pairwise edge overlap was low (Jaccard, 0.06–0.11). Against a 151-triple dual-annotated gold set, expert precision was 0.914 (95% CI = 0.858–0.949) with overlapping per-model intervals, while an LLM judge systematically under-rated precision on the 51 triples it scored (raw agreement, 0.65). External support rose with cross-model consensus, but naive majority agreement did not beat the best single model (DeepSeek, 0.377); only unanimous four-model agreement exceeded it (0.423, + 12% relative) at ~ 4% edge coverage. The unanimity interval (95% CI = 0.362–0.486) overlaps that of the best single model (0.358–0.397), so unanimity is best read as a conservative reliability filter rather than a general fix for graph quality. Consensus is a tunable precision–coverage control, not a free-lunch gain; under the conditions tested here—four models, one domain, one prompt, and schema—the choice of extraction model mattered more than naive aggregation.

## Introduction

Immune-checkpoint blockade has reshaped oncology over the past decade. Antibodies against CTLA-4 and the PD-1/PD-L1 axis produce durable responses across melanoma, lung cancer, and many other tumor types (1–6). The clinical repertoire has expanded from single agents to combinations and to newer targets such as LAG-3, alongside a growing set of response biomarkers (7–13). Each advance adds primary reports, trials, and mechanistic studies, so the checkpoint literature now grows faster than any individual can read. Structuring this evidence into a computable form is a standing problem for the field.

Biomedical knowledge graphs address that problem by encoding entities and their relations as nodes and edges, supporting integration, query, and computational reasoning (14). Curated and text-mined graphs such as Hetionet, PrimeKG, and GNBR have enabled drug repurposing, polypharmacy prediction, and hypothesis generation (15–19). Building such graphs has historically required either expert curation or bespoke relation-extraction pipelines, both of which are costly to develop and to maintain as the literature moves. Once built, graphs are embedded into vector spaces so that missing edges can be scored, and link prediction over these embeddings has been used to rank repurposing and mechanistic hypotheses (20–25). The same structured evidence underpins computational target and compound prioritization more broadly, from structure-based derivative design with ADME and toxicity prediction to pharmacophore-based virtual screening refined by molecular-dynamics simulation (26, 27). The individual mechanistic claims that such pipelines consume—that silencing a gene alters sensitivity to therapy, or that a delivered factor activates a signaling pathway—are established one experiment at a time (28, 29), and relations of exactly these kinds are what the schema used here asks each model to extract.

Large language models (LLMs) offer a general-purpose alternative. Instruction-tuned models extract entities and relations from free text with little or no task-specific training (30–35), and encode substantial clinical and biomedical knowledge (32, 36, 37). This convenience carries a cost that is easy to overlook. LLMs generate fluent output that can be factually wrong, and different models—trained on different data and objectives—may read the same sentence differently (38). When an LLM is used as the extraction engine for a KG, these idiosyncrasies propagate into the graph’s topology and into any downstream inference. Most published LLM-to-KG pipelines rely on a single model and report no cross-model agreement, so the reproducibility of the resulting graph is unknown.

Here we treat the choice of extraction model as an experimental variable rather than a fixed component. We build four independent checkpoint KGs from one corpus, using four contemporary LLMs (DeepSeek, General Language Model (GLM), Kimi, and Qwen) (39–41) under identical conditions. We chose the immune-checkpoint literature for three reasons. It is a large, fast-moving, and clinically consequential corpus in which automated synthesis is actively pursued; its core vocabulary is compact and largely unambiguous—roughly two dozen checkpoint genes and a well-defined set of approved antibodies—so that disagreement between models is attributable to the models rather than to entity ambiguity; and it is densely covered by curated external resources, which supplies an independent yardstick against which extracted edges can be checked. We then quantify how far they diverge and whether combining them helps. We evaluate the graphs along five axes: extraction landscape, triple correctness against human experts with a calibrated LLM judge, topological and embedding divergence, and external validation. We also test a consensus protocol that trades precision against coverage. This study deliberately abstains from immunotherapy-response prediction on patient cohorts, focusing instead on the upstream question of whether knowledge extracted by LLMs is consistent enough to be trusted.

## Results

### A multi-LLM benchmark for checkpoint knowledge-graph construction

**Figure 1:**
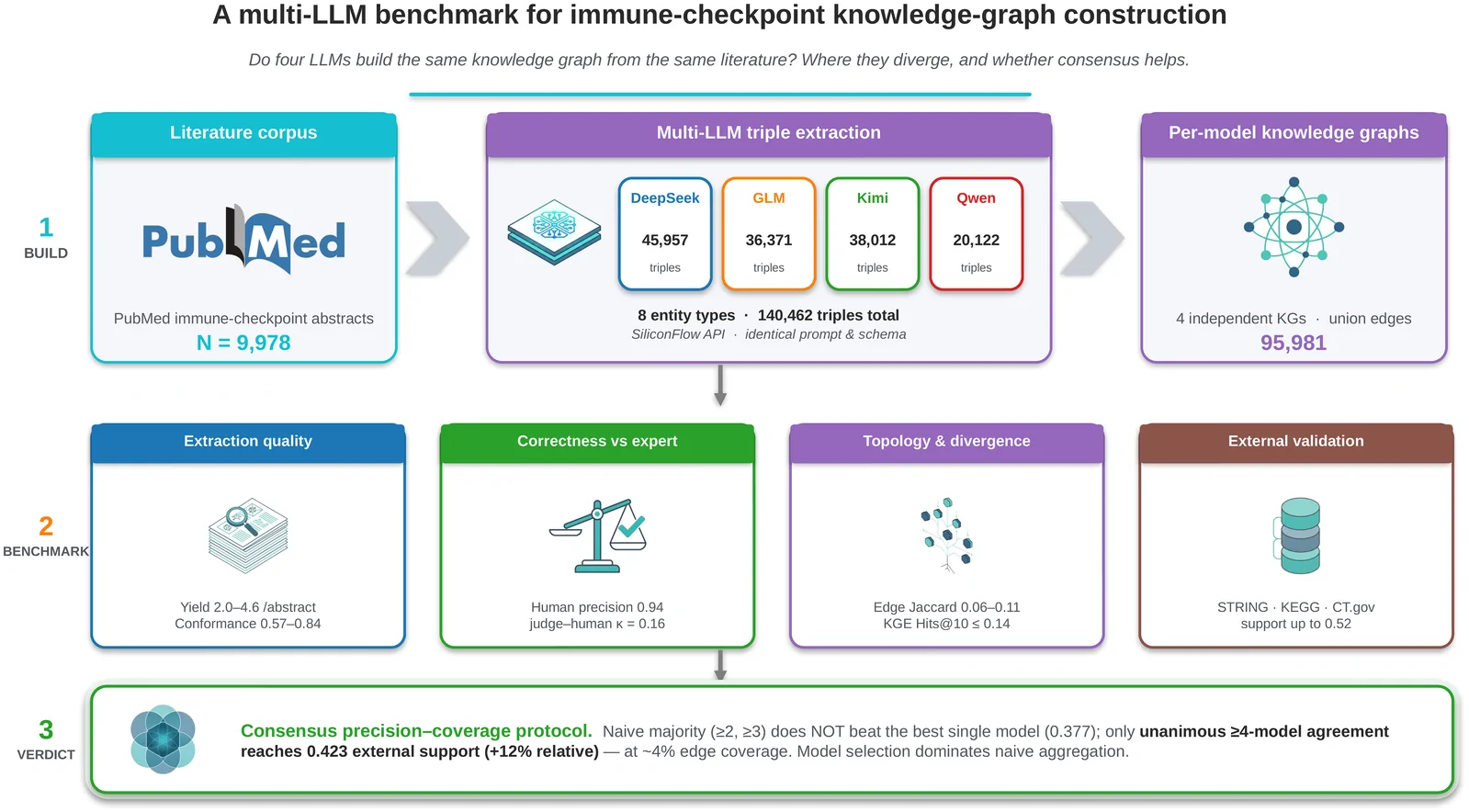
A multi-LLM benchmark for immune-checkpoint knowledge-graph construction. A PubMed immune-checkpoint corpus (*N* = 9,978 abstracts) is passed through four LLMs (DeepSeek, GLM, Kimi, and Qwen) under an identical prompt and eight-entity-type schema, yielding 140,462 triples that form four per-model knowledge graphs (with a union of 95,981 edges). Five evaluation axes (**Figures 2–5**) probe extraction quality, correctness against a human expert, topological and embedding divergence, and external validation under a consensus protocol.

**Figure 2:**
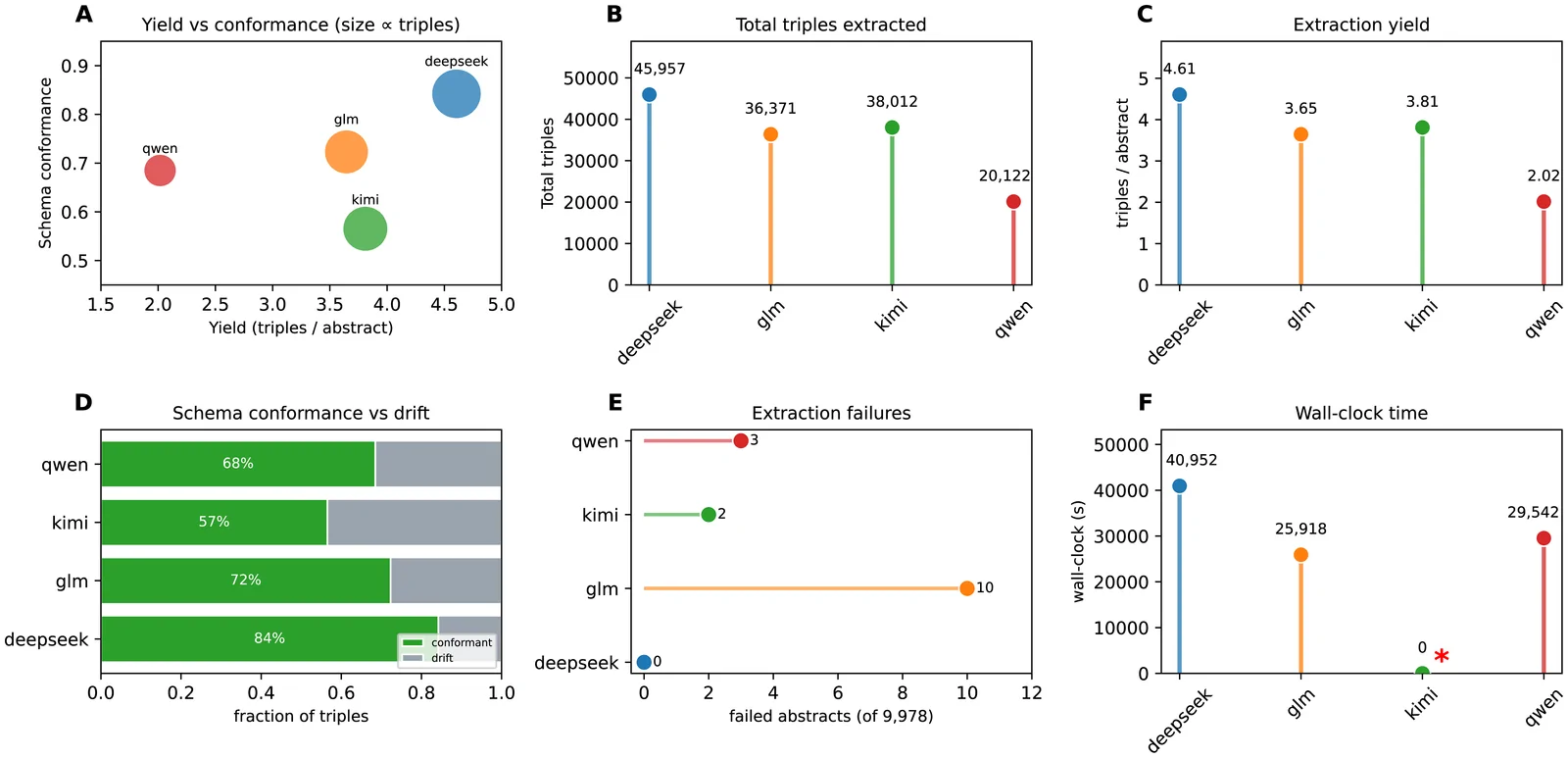
Extraction landscape across four LLMs (*N* = 9,978 abstracts). **(A)** Yield (triples per abstract) versus schema conformance; bubble area is proportional to total triples. **(B)** Total triples per model. **(C)** Extraction yield. **(D)** Schema-conformant versus schema-drift fraction as a 100%-stacked distribution. **(E)** Extraction failures (abstracts producing no parseable output, out of 9,978). **(F)** Wall-clock time. Kimi’s near-zero value in **(F)** is a timing artefact of a checkpointed run whose elapsed time was not recorded. It is flagged with a red asterisk and a footnote in the panel and must be disregarded as a speed measure rather than read as a speed advantage.

**Figure 3:**
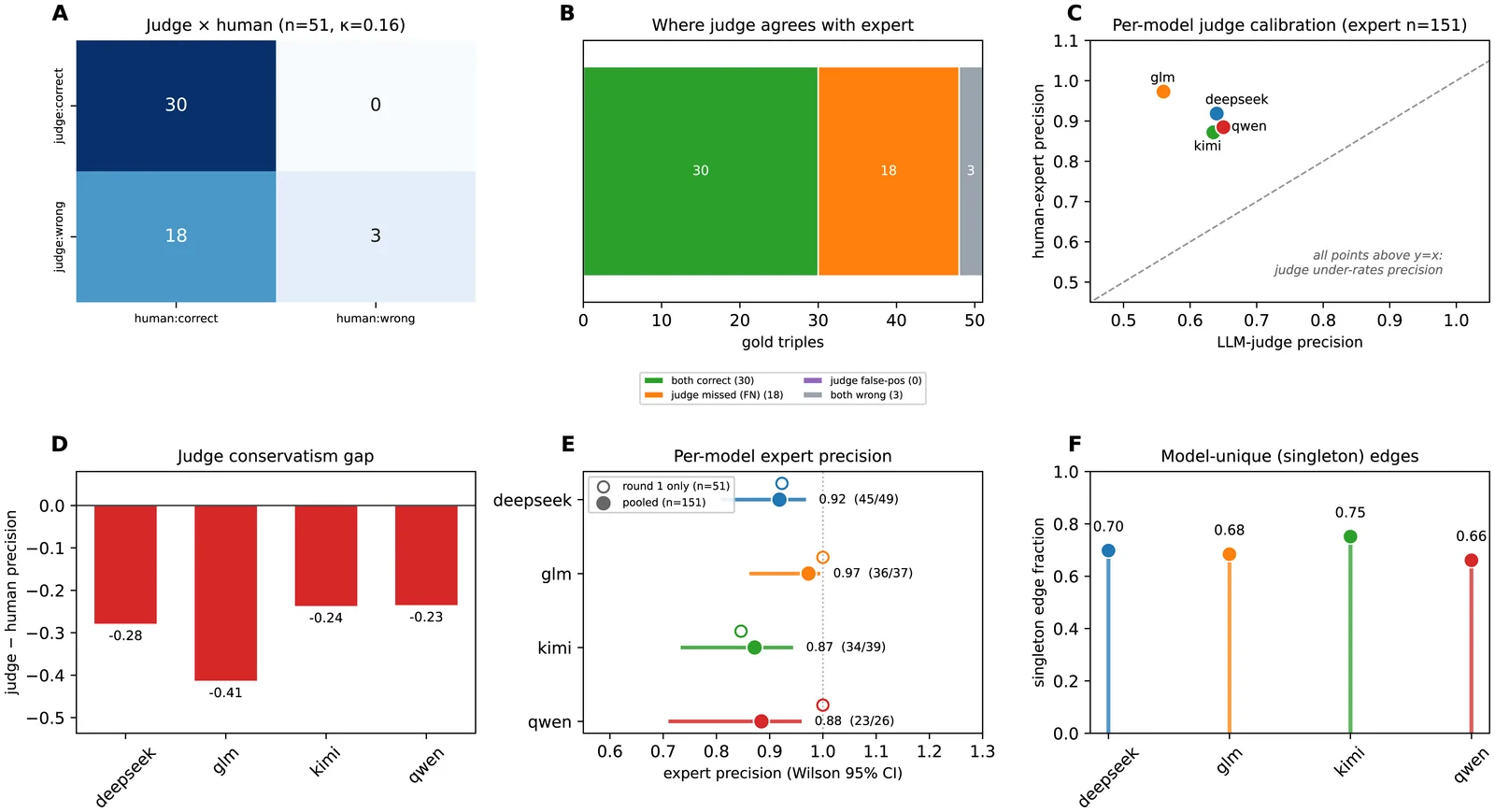
Triple correctness and LLM-judge calibration. **(A)** Confusion matrix of the LLM judge (DeepSeek-V3) against the human expert over 51 gold triples (raw agreement = 0.65, Cohen’s κ = 0.16). **(B)** Breakdown of the 51 gold triples by judge × human outcome (both correct, 30; judge false-negative, 18; judge false-positive, 0; both wrong, 3). **(C)** Per-model LLM-judge versus human-expert precision; all points lie above the identity line. **(D)** Judge-minus-human precision gap per model (all negative). **(E)** Human-expert precision per model with annotation counts. **(F)** Model-unique (singleton) edge fraction. The gold set (*n* = 51) was labelled by two experts; agreement rose from raw 55% (κ = 0.05) to 92% (PABAK = 0.84) after both were re-scored under a written rubric. Cohen’s κ = 0.29 is deflated by a 48:3 class imbalance (the kappa paradox) and is read alongside raw agreement and PABAK. PABAK, prevalence-adjusted bias-adjusted kappa. **(A, B)** The 51 triples the LLM judge scored. **(C–E)** use the pooled expert gold set of 151 triples (51 from round 1 plus 100 from the expansion); in **(E)**, filled markers with bars are the pooled estimate with its Wilson 95% confidence interval, and hollow markers are the round-1-only estimate, showing that the two 1.00 values observed in round 1 did not survive the larger sample. The four intervals overlap, so **(C–E)** do not rank the models.

**Figure 4:**
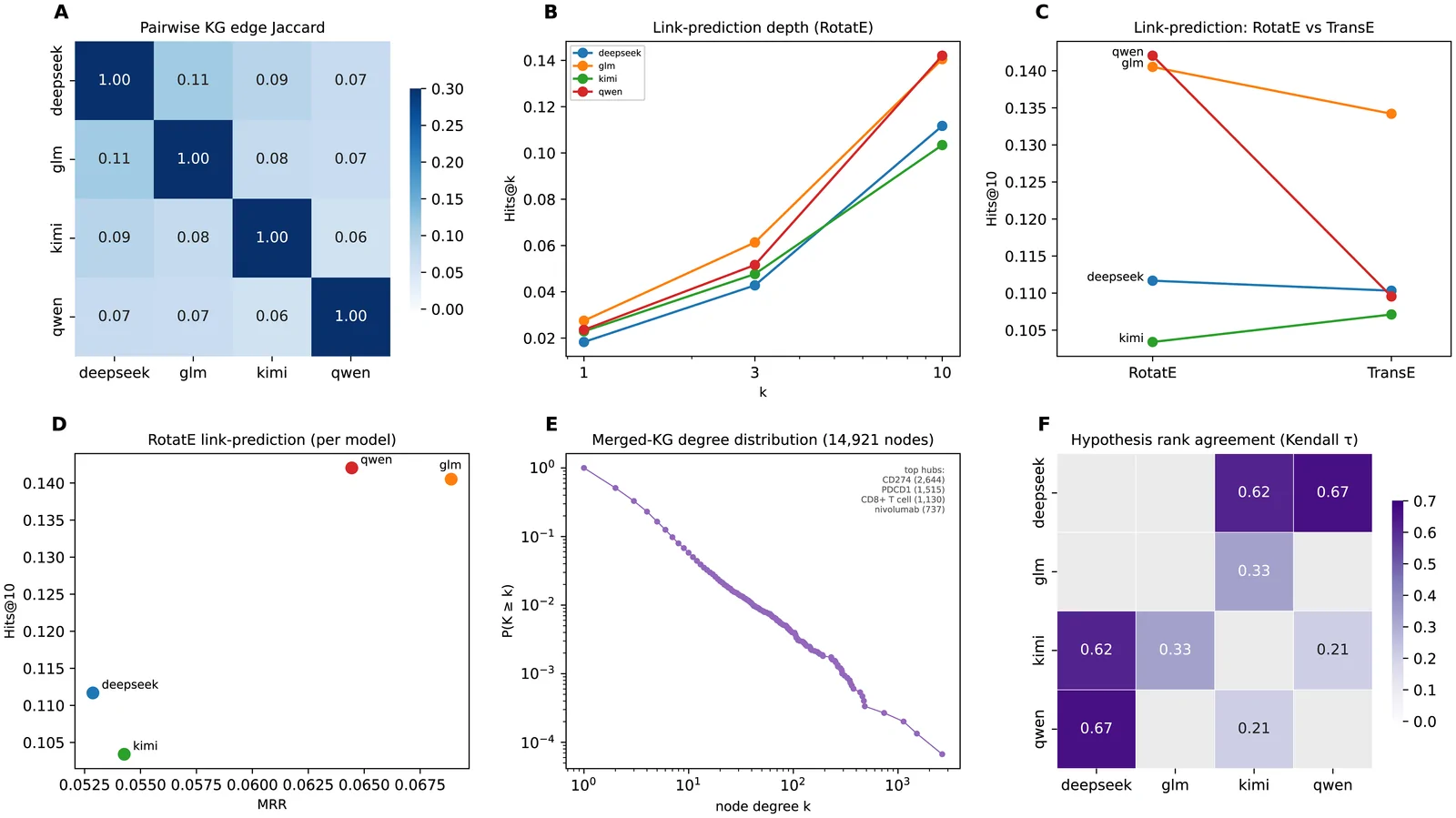
KG topology, embedding link prediction, and hypothesis divergence. **(A)** Pairwise KG edge Jaccard (0.06–0.11). **(B)** RotatE link-prediction ranking depth (Hits@1/3/10) per model. **(C)** RotatE versus TransE Hits@10. **(D)** RotatE MRR versus Hits@10 per model. **(E)** Degree distribution (CCDF, log–log) of the merged KG (14,921 nodes, 33,110 edges): scale-free with a heavy tail (49% of nodes have degree 1); top hubs are CD274/PD-L1 (degree 2,644), PDCD1/PD-1 (1,515), CD8+ T cell (1,130), and nivolumab (737). **(F)** Pairwise Kendall τ of shared-hypothesis rankings (grey = too few shared hypotheses). Link prediction is weak for every model (Hits@1 ≈ 0.00–0.03, Hits@10 < 0.15); **(B–D)** are exploratory, quantifying cross-model divergence rather than embedding capability, and the full training and evaluation protocol is given in **Supplementary Table 8**. MRR, mean reciprocal rank; CCDF, complementary cumulative distribution function.

**Figure 5:**
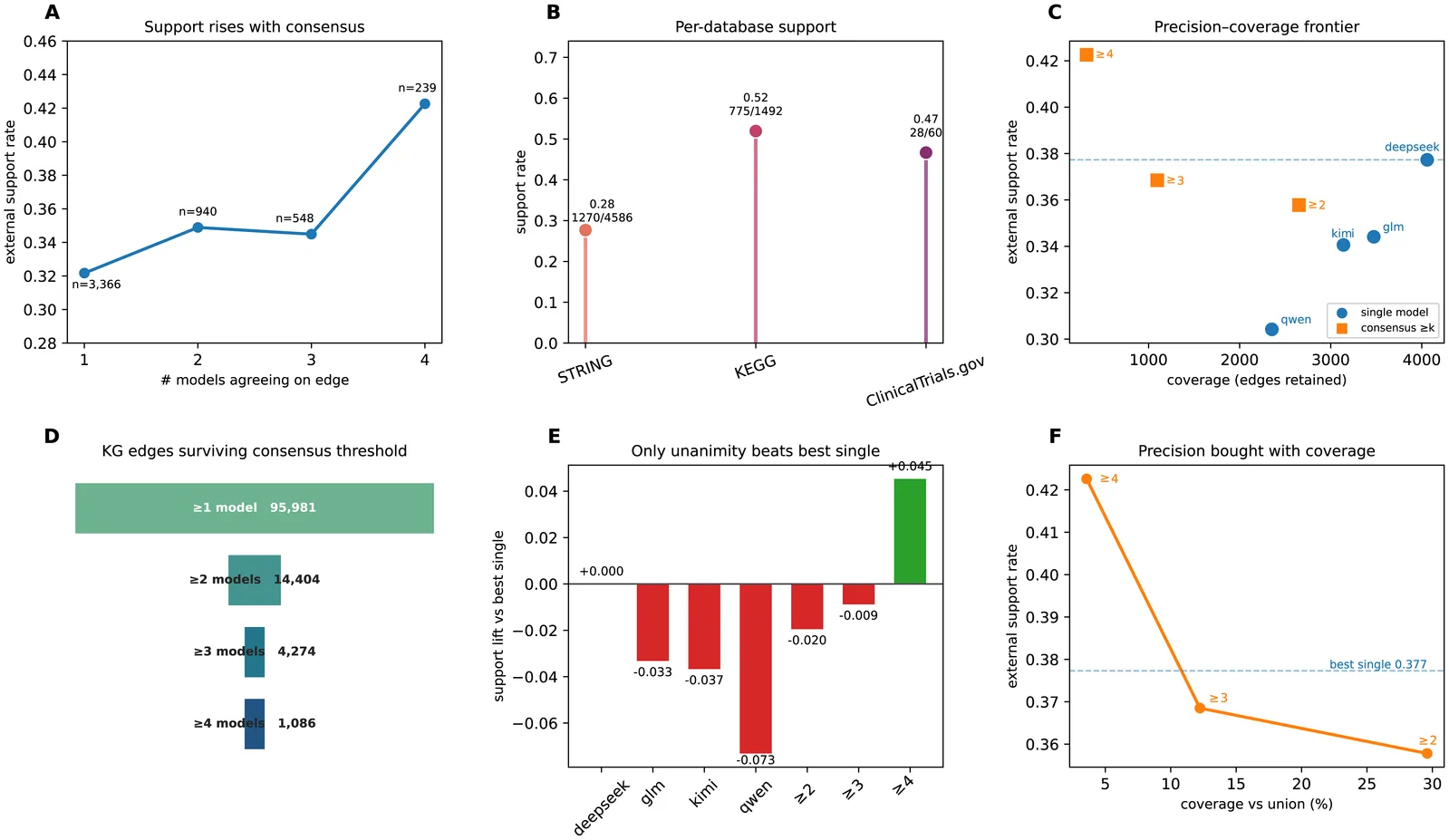
External validation and the consensus precision–coverage protocol. **(A)** External support rate rises with cross-model consensus level. **(B)** Per-database support: STRING 0.28, KEGG 0.52, and ClinicalTrials.gov 0.47. **(C)** Precision–coverage frontier: every single model versus ≥ k-consensus KGs. **(D)** Edge survivorship funnel: union edges retained at each consensus threshold (95,981 → 14,404 → 4,274 → 1,086). **(E)** Support lift over the best single model (DeepSeek, 0.377) per strategy. **(F)** Precision achieved with coverage across consensus thresholds. Naive majority consensus (≥ 2, ≥ 3) does not beat the best single model; only unanimous ≥ 4 agreement exceeds it (0.423, + 12% relative) at ~ 4% edge coverage. Wilson 95% confidence intervals for every stratum are provided in **Supplementary Table 9**; the ≥ 4 interval (0.362–0.486) overlaps DeepSeek’s (0.358–0.397), so unanimity is a conservative reliability filter rather than a demonstrated improvement.

We assembled a corpus of 9,978 immune-checkpoint PubMed abstracts and processed it through four LLMs—DeepSeek, GLM, Kimi, and Qwen (**Figure 1**). All four were served through a single API, with one shared prompt and an eight-entity-type schema (Checkpoint, Cancer, Drug, CellType, Gene, Pathway, Biomarker, and ClinicalTrial). The four models produced 140,462 triples in total, which we assembled into four per-model knowledge graphs and one union graph of 95,981 edges. Five evaluation axes then probed each graph in turn: the extraction landscape (**Figure 2**), correctness against a human expert (**Figure 3**), topological and embedding divergence (**Figure 4**), and external validation under a consensus protocol (**Figure 5**). Holding the corpus, prompt, and schema fixed isolates the model itself as the only source of variation between graphs.

### Extraction volume and schema conformance diverge across models

The four models extracted markedly different numbers of triples from the same 9,978 abstracts (**Figures 2A–C**; **Supplementary Table 1**). DeepSeek returned the most, 45,957 triples (4.61 per abstract), followed by Kimi (38,012; 3.81) and GLM (36,371; 3.65). Qwen was the most conservative, with 20,122 triples (2.02 per abstract)—a 2.3-fold spread in yield. Parseable-output failures were rare for every model: 0 abstracts for DeepSeek, two for Kimi, three for Qwen, and 10 for GLM, all below 0.1% (**Figure 2E**).

Schema conformance—the fraction of a model’s triples whose head and tail entities both fall within the eight permitted entity types—separated the models more sharply than yield (**Figure 2D**). DeepSeek’s triples were 84.2% conformant, GLM’s 72.3%, and Qwen’s 68.5%, but only 56.5% of Kimi’s triples conformed, meaning Kimi drifted off-schema on 43.5% of its output. Higher yield did not track higher conformance: Kimi ranked second in volume but last in conformance, so the two dimensions capture different failure modes (**Figure 2A**). Wall-clock time ran to 25,918–40,952 s for DeepSeek, GLM, and Qwen (**Figure 2F**); Kimi’s near-zero value is a timing artefact of a checkpointed run whose elapsed time was not recorded and does not indicate a speed advantage. The bar is flagged with an asterisk in **Figure 2F** and should be disregarded as a speed measure.

### Triple correctness and a systematically conservative LLM judge

To assess whether extracted triples are actually correct, we scored a gold set of 51 triples against a human expert and against an LLM judge (DeepSeek-V3). In the first annotation round, human-expert precision was high for every model: 1.00 for GLM and Qwen (*n* = 13 and 12), 0.92 for DeepSeek (*n* = 13), and 0.85 for Kimi (*n* = 13) (**Figure 3E**; **Supplementary Table 2**). As 12–13 triples per model support no useful comparison, we expanded the gold set by a further 100 triples scored by the same two experts, giving 151 in total. Pooled expert precision was 0.914 (138/151, Wilson 95% confidence interval [CI] = 0.858–0.949) under the strict adjudication rule and 0.954 (144/151, 0.907–0.977) under the lenient one. Per model, the pooled values are GLM ay 0.973 (36/37, 0.862–0.995), DeepSeek at 0.918 (45/49, 0.808–0.968), Qwen at 0.885 (23/26, 0.710–0.960), and Kimi at 0.872 (34/39, 0.733–0.944) (**Supplementary Tables 2**, **S11**; **Figure 3E**). The apparent 1.00 precision of the first round did not survive the larger sample, and the four intervals still overlap, so the correctness axis establishes no ranking between models—only that individual triples are usually well formed for all four. Individual triples were usually well-formed; the divergence between models lies in which triples they choose to emit, not in gross error rates.

The LLM judge told a different and more cautious story. Against the human expert over the 51 gold triples, the judge reached raw agreement of 0.65 with Cohen’s κ = 0.16 (**Figure 3A**). The disagreement was one-sided (**Figure 3B**). Of the 51 triples, the judge and the expert both accepted 30 and both rejected three. The judge rejected 18 triples the expert had accepted, and never falsely accepted one (0 false positives). Judge precision estimates therefore sat below the human estimates for every model, by 0.21 (Kimi) to 0.44 (GLM) (**Figures 3C, D**). An LLM judge here is conservative, not lenient: it under-rates precision rather than rubber-stamping the extractor’s output. Two caveats bound this reading. The judge (DeepSeek-V3) belongs to the same model family as one of the four extraction conditions and is therefore not independent of it, although it did not favor that condition—Qwen received the highest judged precision (**Supplementary Table 2**). Moreover, only three of the 51 gold triples were adjudicated incorrect, so the judge’s false-positive behavior is essentially untestable here; the zero false-positive count is gold-set-specific and preliminary rather than a general property.

Human annotation itself required an explicit standard. Two experts independently scored the gold set; a first round agreed on only 55% of triples (Cohen’s κ = 0.05), reflecting a systematic strictness gap rather than random noise. After both experts rescored the set under a written rubric, agreement rose to 92% (prevalence-adjusted bias-adjusted kappa [PABAK] = 0.84) with four residual disagreements, which were adjudicated by applying the rubric clause by clause (**Supplementary Table 3**). All four resolutions coincided with annotator A, but the pooled estimate is invariant to this: resolving all four the other way also yields 48 of 51 correct (0.941) and moves individual models by at most one triple. On the 100 newly annotated triples, scored under the same rubric but never seen when it was written, the two experts agreed on 94 (Cohen’s κ = 0.54, PABAK = 0.88; **Supplementary Figure 3A**). The rubric therefore transfers: the jump from 55% to 92% agreement in round 1 was not an artefact of reconciling the same triples twice. Cohen’s κ = 0.29 on the reconciled set is deflated by the 48:3 correct-to-wrong class imbalance—the kappa paradox—and must be read alongside raw agreement and PABAK. The fact that a shared rubric moved two experts from κ = 0.05 to 92% agreement is itself informative: LLM-triple correctness is judgeable, but only against an explicit standard.

Beyond correctness, the four graphs were largely made of private edges. The model-unique (singleton) fraction—the proportion of a model’s edges that, after normalization, appear in exactly one of the four per-model graphs and are therefore produced by no other model—ranged from 0.66 (Qwen) to 0.75 (Kimi) (**Figure 3F**; **Supplementary Table 2**). Two-thirds to three-quarters of each model’s graph were unique to that model, foreshadowing the low overlap quantified next.

### Topological and embedding divergence between the four graphs

The four graphs barely intersect. Pairwise edge Jaccard similarity ranged from 0.06 to 0.11 (**Figure 4A**): the most similar pair (DeepSeek–GLM, 0.11) still shared only about one edge in nine, and the least similar (Kimi–Qwen, 0.06) about one in sixteen. Four models reading the same 9,978 abstracts thus wrote four almost-disjoint graphs.

We trained knowledge-graph embeddings (RotatE and TransE) on each graph and evaluated link prediction (**Figures 4B–D**; **Supplementary Table 4**). Absolute accuracy was low for every model and both embeddings: RotatE Hits@1 fell between 0.018 and 0.028, and Hits@10 was below 0.15, with TransE lower still. These panels are therefore exploratory: they compare models against one another and are not a claim that any graph supports strong link prediction. The full training and evaluation protocol—splits, negative sampling, filtered ranking, hyperparameters, and random seed—is specified in **Supplementary Table 8**. RotatE outperformed TransE on Hits@10 for each model (**Figure 4C**), and the between-model ranking was consistent across the two embeddings.

To confirm that the union graph is at least biologically sensible, we examined the degree distribution of the merged KG (14,921 nodes, 33,110 edges after node harmonization) (**Figure 4E**). The distribution is heavy-tailed and approximately scale-free, with 49% of nodes at degree 1. The highest-degree hubs are the canonical checkpoints: CD274/PD-L1 (degree 2,644), PDCD1/PD-1 (1,515), CD8+ T cell (1,130), and nivolumab (737). The union graph therefore has the topology expected of a real biomedical network, even though the individual models that built it disagree. The models also diverged in what they proposed: top-k hypothesis overlap between model pairs was near zero (only DeepSeek–Kimi shared 1 of the top 50; **Supplementary Figure 2**), and rank correlation of shared hypotheses (Kendall τ) rested on too few common items (*n* = 2–26) to be more than indicative (**Figure 4F**).

### External validation and a consensus precision–coverage protocol

We validated edges against external resources (Search Tool for the Retrieval of Interacting Genes/Proteins (STRING), Kyoto Encyclopedia of Genes and Genomes (KEGG), and ClinicalTrials.gov) and asked whether combining models improves reliability. Support rates differed by database: 0.52 of checkable edges were supported in KEGG, 0.47 in ClinicalTrials.gov, and 0.28 in STRING (**Figure 5B**). Among single models, external support ranged from 0.304 (Qwen) to 0.377 (DeepSeek) (**Figure 5C**; **Supplementary Table 5**), with DeepSeek the strongest single extractor by this measure. Checkable edge classes, mapping rules, denominators, and Wilson 95% confidence intervals for every stratum are given in **Supplementary Table 9**. Support rate measures agreement with a reference database and is not a measure of biological correctness: a correct novel interaction that is absent from STRING or KEGG counts as unsupported, and a co-pathway gene pair can count as supported even when the specific directed claim is wrong.

External support did rise with cross-model consensus, but the gain was modest and nonmonotonic in value (**Figures 5A, E**). Edges endorsed by at least two models reached 0.358 support, at least three 0.369, and all four 0.423 (**Supplementary Table 5**). Only unanimous four-model agreement exceeded the best single model. The ≥ 2 and ≥ 3 thresholds actually fell below DeepSeek’s 0.377, by 0.020 and 0.009, respectively; ≥ 4 gained 0.046, a +12% relative lift (**Figure 5E**). That gain came at a steep coverage cost. Requiring consensus stripped the union graph from 95,981 edges to 14,404 (≥ 2), 4,274 (≥ 3), and 1,086 (≥ 4) (**Figure 5D**), so the ≥ 4 threshold retained about 4% of checkpoint edges (**Supplementary Table 5**). Plotting precision against coverage makes the trade-off explicit: consensus buys precision only by discarding most edges, and no threshold both beats DeepSeek and preserves broad coverage (**Figures 5C, F**). The unanimity gain is also small relative to its uncertainty: the ≥ 4 interval (95% CI 0.362–0.486) overlaps DeepSeek’s (0.358–0.397). Unanimity is therefore best described as a conservative reliability filter that enriches for canonical, well-documented relations, not as a general improvement in graph quality (**Supplementary Table 9**). Naive majority consensus is not a free-lunch improvement; it is a tunable precision–coverage control, and in this setting, selecting a strong single model outweighed naive aggregation.

To make these categories concrete, we audited individual edges spanning four outcomes (**Supplementary Table 10**): expert-accepted triples, expert-rejected triples, edges asserted by the abstracts and reproduced by several models yet absent from STRING and KEGG, and unanimous (*k* = 4) edges with and without external support. The audit also exposes a structural limitation of the benchmark: none of the 51 gold triples falls inside the STRING/KEGG-checkable gene–gene subgraph, so the correctness axis and the external-validation axis measure different edge populations and cannot be substituted for one another.

## Methods

### Corpus construction

Immune-checkpoint abstracts were retrieved from PubMed and de-duplicated to a working corpus of 9,978 abstracts. The corpus was fixed before any extraction so that all four models received identical input. Only title and abstract text were used; no full-text or figure content was included.

### LLM triple extraction

Four instruction-tuned LLMs were used: DeepSeek (deepseek-ai/DeepSeek-V3), GLM (THUDM/GLM-4-32B-0414) and Qwen (Qwen/Qwen2.5-32B-Instruct), all served through the SiliconFlow inference API (https://docs.siliconflow.cn), and Kimi (moonshot-v1-8k), served through the Moonshot API (https://platform.moonshot.cn/docs) because that model is not offered by SiliconFlow. All extractions ran between 7 and 9 July 2026. Decoding was identical across models (temperature 0.1; max_tokens 4,096, raised to 6,000 for Kimi; 10 concurrent requests). JSON output was enforced with response_format = {“type”: “json_object”} for the three SiliconFlow models; moonshot-v1-8k does not support that parameter, so JSON was enforced at the prompt level and parsed with the same parser. Each call was retried up to three times with exponential back-off, and a call that still failed to yield parseable JSON was recorded as an extraction failure. Exact identifiers, endpoints, context limits, and settings are listed in **Supplementary Table 6**. We first attempted the frontier reasoning variants of these families, but in preflight theyneeded 600–1,000 s per abstract, which made processing a 9,978-abstract corpus four times infeasible, and one of them returned no triples at all; we therefore used the fast instruct variants above, which is a scope condition on the conclusions. Each model received the same extraction prompt and the same schema of eight entity types (Checkpoint, Cancer, Drug, CellType, Gene, Pathway, Biomarker, and ClinicalTrial); relations were extracted as (head, relation, and tail) triples. Prompt, schema, and decoding settings were identical across models; the model identifier was the only variable. Outputs that could not be parsed into the triple format were counted as extraction failures (**Supplementary Table 1**). Per-model triple counts, yield, schema conformance, and wall-clock time were logged directly from the pipeline.

### Knowledge-graph assembly

Extracted triples were normalized and assembled into one directed graph per model, and into a union graph over all four models (95,981 edges). Entity strings were harmonized before edges were merged: surface forms were lower-cased for lookup, checkpoint and gene aliases were mapped to HGNC symbols through a curated dictionary covering 25 checkpoint genes and their aliases, drug brand names were mapped to INN generic names, and cancer-type abbreviations were expanded. Relations were restricted to the 13 predefined labels; any other relation string was recorded as schema drift and excluded. An edge is the triple (canonical head, relation label, canonical tail), and two edges are identical if and only if all three components match, the source PMID being edge metadata rather than part of the edge key. Symmetric relations are stored once with sorted endpoints so that (A, r, B) and (B, r, A) collapse to one edge; self-loops arising after normalization are dropped; duplicates within a model are collapsed while the set of models that produced an edge is retained and defines its consensus level k. The complete rule set, with before-and-after examples, is given in **Supplementary Table 7**. The merged graph used for the degree analysis contained 14,921 nodes and 33,110 edges. Schema conformance was defined as the fraction of a model’s triples whose head and tail both fell within the eight permitted entity types; the complement was reported as schema drift.

### Gold-standard annotation and inter-annotator agreement

A gold set of 51 triples was sampled across models and scored independently by two experts (annotator A, an oncology-trained PhD physician; annotator B, a second expert) as correct or incorrect. A first annotation round showed low agreement (raw 55%, Cohen’s κ = 0.05) driven by a systematic difference in strictness. Both annotators then rescored the set under a written rubric (ANNOTATION_GUIDELINE.md); agreement rose to raw 92% (PABAK = 0.84, Cohen’s κ = 0.29). Because Cohen’s κ is deflated by the 48:3 class imbalance—the kappa paradox—raw agreement and PABAK are reported alongside it (42–44). Both annotators scored independently in both rounds, without sight of each other’s labels or of the LLM judge’s verdict, which was withheld from the annotation sheet to avoid anchoring. The identity of the extraction model was, however, visible in the sheet; we did not blind it, and we note this as a limitation. As the rubric asks only whether the cited sentence supports the triple, we do not expect model identity to drive the label, but we cannot exclude it. The four residual disagreements were adjudicated by applying the rubric clause by clause rather than by seniority, and all four resolutions coincided with annotator A. To avoid potentially collapsing the gold standard onto a single expert’s standard, we checked the alternative: resolving all four in favor of annotator B gives the same pooled precision (48 of 51, 0.941) and changes per-model precision by at most one triple (**Supplementary Table 3**). A gold standard was then expanded. A further 100 triples were drawn from the same candidate pool under the same stratified dual-annotator protocol, in the same sheet layout and under the same conditions, and scored independently by the same two experts, giving a pooled set of 151 triples. Round-2 agreement was 0.94 raw (94 of 100; Cohen’s κ = 0.54, PABAK = 0.88) with six disagreements, against 0.55 raw before the rubric existed in round 1, indicating that the rubric transfers to unseen triples rather than to the triples it was written on. The six round-2 disagreements were not adjudicated; instead, every round-2 number is reported under two prespecified rules that bracket any adjudication—strict (correct only if both annotators agreed) and lenient (correct if either did)—so the conclusions can be checked against the worst case (**Supplementary Table 11**; **Supplementary Figure 3**). Per-model precision attributes each gold triple to one producing model; where several models produced the same triple, it is assigned to one of them by a deterministic hash of the triple, so that no model is systematically favored. Counting a shared triple once for every producer instead gives DeepSeek at 0.906, GLM at 0.978, Kimi at 0.906, and Qwen at 0.872, with the same overlapping intervals and the same conclusion.

### LLM-as-judge scoring

An LLM judge (DeepSeek-V3) scored each gold triple as correct or incorrect using the same rubric provided to the human annotators (45). Judge output was compared with the reconciled human gold standard by a 2 × 2 confusion matrix, raw agreement, and Cohen’s κ (**Figures 3A, B**; **Supplementary Table 3**). Per-model judge precision and human-expert precision were computed on the annotated subsets, and their difference reported as the judge-conservatism gap (**Figures 3C–E**; **Supplementary Table 2**). The judge shares a model family with one of the four extraction conditions and is therefore not independent of it, and the 48:3 class balance of the gold set leaves its false-positive behavior untestable; judge-derived precision is accordingly reported as a gold-set-specific lower bound rather than as ground truth.

### Knowledge-graph embedding and link prediction

Each per-model graph was embedded with RotatE and TransE (20, 21). Link prediction was evaluated under the filtered protocol by ranking candidate heads and tails and reporting Hits@1, Hits@3, Hits@10, and mean reciprocal rank (MRR) (**Supplementary Table 4**). Triples were split 80/10/10 into training, validation, and test sets, the training fraction being raised in steps to 0.85, 0.90, or 0.95, where a random split left entities uncovered. Embedding dimension was 128; training ran for 200 epochs with batch size 1,024, Adam at learning rate 1*e*−3, and 10 uniformly corrupted negatives per positive, under a single random seed (42) applied identically to all four graphs, with no hyperparameter search. The full protocol is given in **Supplementary Table 8**. Metrics are used to compare models with one another; absolute values are low for all models and are not interpreted as evidence of strong predictive capability. Generated hypotheses (top-ranked missing edges) were compared across model pairs by top-k overlap (*k* = 10, 20, 50; **Supplementary Figure 2**) and by Kendall τ over shared hypotheses (**Figure 4F**).

### External validation and consensus protocol

Checkable edges were validated against three external resources: STRING for protein–protein associations (46), KEGG for pathway relations (47), and ClinicalTrials.gov for drug–disease trial relations (48). Validation was restricted to gene–gene edges carrying an interaction-like relation. An edge was checkable against STRING if at least one endpoint was one of the 25-panel checkpoint genes, in which case the complete STRING neighborhood of that gene (interaction_partners, combined score ≥ 400) was retrieved so that a nonneighbor is a trustworthy negative. An edge was checkable against KEGG if both endpoints lay within the 1,272-gene union of 11 checkpoint-related KEGG pathways and was supported if the two genes shared at least one of those pathways. ClinicalTrials.gov was queried edge by edge on a capped sample of unique drug–cancer edges and is reported separately. The support rate is the number of supported divided by checkable edges; denominators and Wilson 95% confidence intervals for every stratum are reported in **Supplementary Table 9**, together with the false-positive and false-negative mechanisms that prevent the support rate from being read as a precision estimate. Consensus KGs were formed by retaining edges endorsed by at least *k* models (*k* = 2, 3, 4). Each consensus KG was compared against every single model on external support rate and on coverage relative to the union graph (**Figure 5**; **Supplementary Table 5**).

### Statistical analysis and reproducibility

Agreement was quantified with raw agreement, Cohen’s κ, and PABAK; the degree distribution was examined on log–log axes as a complementary cumulative distribution. All reported numbers are produced directly by the analysis pipeline from the extraction and validation outputs, and figure values were checked against the source tables before release. Binomial proportions—precision and external-support rates—are reported with Wilson score 95% confidence intervals, which behave better than normal-approximation intervals at the small denominators and extreme proportions that occur here.

## Discussion

Four large language models, given the same 9,978 abstracts, the same prompt, and the same schema, produced four knowledge graphs that scarcely overlap. Yield spanned 2.3-fold, schema conformance ranged from 57% to 84%, and pairwise edge Jaccard sat between 0.06 and 0.11, with two-thirds to three-quarters of each graph unique to its model. The disagreement is not mainly a matter of individual triples being wrong—human-expert precision was 0.85–1.00 for every model—but of which relations each model chooses to surface. For anyone building a biomedical KG with a single LLM under conditions like ours, that model choice silently determines most of the graph.

This finding sits alongside a body of work using LLMs for biomedical extraction and KG construction (30–35), most of which reports single-model pipelines without cross-model agreement. Our results argue that agreement should be measured rather than assumed. The pattern is consistent with the reproducibility concerns raised more broadly for machine-learning-driven science (49), and it extends them from model training to model-as-annotator. Where curated resources such as Hetionet and PrimeKG derive their authority from explicit provenance (15, 16), an LLM-built graph inherits the idiosyncrasies of its extractor, which our benchmark makes visible.

Two secondary findings bear on how such graphs should be evaluated. First, the LLM judge was systematically conservative. It agreed with human experts only 65% of the time. When it disagreed, it rejected triples the experts had accepted rather than the reverse (18 false negatives, 0 false positives). An LLM judge is a useful but biased instrument, and reporting it as a lower bound on precision is safer than treating it as ground truth (45). Second, human agreement itself was fragile until a rubric was imposed—κ rose from 0.05 to a reconciled 92% raw agreement only after both annotators adopted a written standard. The kappa paradox, long documented for imbalanced classes (42–44), is on full display here, and underlines why raw agreement and PABAK belong beside κ. The fact that correctness becomes judgeable only under an explicit rubric is a practical lesson for anyone assembling a gold standard for LLM output.

The consensus analysis tempers a natural expectation. Combining models is often assumed to improve reliability, as it does in classical ensembles and in self-consistency methods for LLM reasoning (50, 51). Here, naive majority consensus did not beat the best single model on external support. The ≥ 2 and ≥ 3 thresholds fell below DeepSeek’s 0.377; only unanimous four-model agreement exceeded it, at 0.423 (+ 12% relative) and ~ 4% edge coverage. Consensus behaves as a precision–coverage dial, not a free improvement, because agreement among divergent extractors is rare and the rare agreed edges are enriched for easy, well-established relations. The unanimity advantage is moreover within the uncertainty of the estimate—its 95% confidence interval overlaps that of the best single model—so unanimity should be offered as a conservative reliability filter, useful where precision matters more than recall, and not as a general solution to knowledge-graph quality. In practice, selecting a strong single model and, where precision is paramount, filtering to unanimous edges is a more honest recipe than pooling all models and hoping the majority is right.

Several limitations bound these conclusions. The gold standard is small (51 triples, two annotators). We therefore expanded it by 100 triples under the same protocol to 151 in total. Pooled expert precision is 0.914 (138/151, 95% CI = 0.858–0.949), and the four per-model intervals still overlap (**Supplementary Table 11**), so the correctness axis supports no ranking between models even at this size: separating four extractors whose precisions lie within a few points of one another would require a gold set an order of magnitude larger. The LLM judge was not re-run on the expansion, so the judge calibration still rests on 51 triples. Enlarging the dual-annotated set, following established gold-standard annotation practice (52), is the priority before firm correctness claims. Link prediction was weak for every model (Hits@1 ≈ 0.00–0.03, Hits@10 < 0.15). The embedding panels therefore quantify cross-model divergence, not embedding capability, and the Kendall τ comparison rests on few shared items (*n* = 2–26). External validation covers three databases—STRING (46), KEGG (47), and ClinicalTrials.gov (48)—and only a checkable subset of edges. Unsupported edges are therefore not necessarily wrong, only unconfirmed by these sources; other resources such as DGIdb (53) or TCGA expression–survival evidence (54) could extend coverage. We studied four models on one biomedical domain through one API; other models, prompts, schemas, or domains may diverge differently. Finally, the timing for one model was not captured and is excluded from any speed interpretation.

### Scope conditions

These conclusions are conditioned on the experimental setting and are not general laws. We tested four contemporary instruction-tuned models in their fast, nonreasoning variants on one biomedical domain, through two commercial inference endpoints, with a single prompt and a single eight-type schema, in one run per model at temperature 0.1. Frontier reasoning variants, other model families, alternative prompts or schemas, other domains, and repeated sampling from the same model may all behave differently. In particular, run-to-run variability within a single model was not measured, so the divergence reported here bounds between-model variation but not the total variation an LLM-built graph inherits. What the study does establish, within these bounds, is that cross-model agreement cannot be assumed and has to be measured.

The immediate application is defensive: report cross-model agreement whenever an LLM builds a KG, and treat a single-model graph as one sample from a wide distribution rather than a canonical structure. Consensus can be tuned toward precision when needed, but at a coverage cost that must be stated. Looking forward, three steps would turn this critical benchmark into routine quality control for LLM-derived knowledge graphs (14, 18). These are a rubric-guided, expanded gold standard; agreement-weighted edge scoring; and extension to additional models and domains. Until then, the safest reading of an LLM-built checkpoint KG is that the model, not the literature, wrote most of it.

## Data Availability

The original contributions presented in the study are included in the article/**Supplementary Material**. Further inquiries can be directed to the corresponding authors. The analysis pipeline, implemented in the checkkg Python package (extraction, knowledge-graph assembly, embedding, benchmarking and consensus analysis) is available at https://github.com/yangfangs/kgbench.

## Supplementary material

Supplementary Figure 1 KGE link-prediction metrics (model × embedding). Heatmap of Hits@1/3/10 and MRR for each model under RotatE and TransE. Absolute accuracy is low for all models (Hits@1 ≈ 0); reported to compare models, not as a capability claim.

Supplementary Figure 2 Top-k hypothesis overlap between models. Overlap fraction of the top-k ranked hypotheses for all six model pairs (k = 10/20/50). Overlap is ≈0 everywhere: DeepSeek–Kimi shares 1 of the top 50 (0.02), and all other pairs share none.

Supplementary Figure 3 Gold-standard expansion (n = 151). (A) Inter-annotator agreement across the three scoring passes: round 1 before the written rubric existed, round 1 after both annotators re-scored under it, and round 2 on 100 triples that were never seen when the rubric was written. Raw agreement, PABAK, and Cohen’s κ are shown together because κ is deflated by the class imbalance. (B) Round-2 expert precision by the number of models that produced the edge, with Wilson 95% confidence intervals and counts; the strata are small, and the intervals overlap, so no trend is claimed. (C) Sensitivity of the pooled estimate to how the six round-2 disagreements are resolved: filled markers are the strict rule (correct only if both annotators agreed), hollow markers the lenient rule (correct if either did). The two rules bracket any adjudication we did not perform.

Supplementary Table 1 full extraction metrics (triples, yield, failures, conformance, drift, wall-clock) for all four models.

Supplementary Table 2 per-model human-expert precision (with n), LLM-judge precision and singleton-edge fraction.

Supplementary Table 3 judge×human confusion, agreement, Cohen’s κ, gold precision, and human–human IAA with annotator provenance.

Supplementary Table 4 KGE link-prediction metrics (Hits@1/3/10, MRR) under RotatE and TransE.

Supplementary Table 5 single-model and ≥k-consensus edge counts, checkable/supported counts, support rate and coverage.

Supplementary Table 6 exact LLM inference configuration: model identifiers, provider endpoints, query dates, decoding parameters, context limits, JSON enforcement, retry policy and concurrency.

Supplementary Table 7 entity and relation normalization rules and the definition of edge identity, each with a before-and-after example taken from the real output.

Supplementary Table 8 knowledge-graph-embedding protocol: library, models, train/validation/test split, negative sampling, filtered ranking, hyperparameters, random seed, relation handling and leakage controls.

Supplementary Table 9 external-validation edge classes and mapping rules, with checkable and supported counts, support rates, Wilson 95% confidence intervals and the false-positive and false-negative limits of every stratum.

Supplementary Table 10 audited edge case studies: expert-accepted, expert-rejected, asserted-but-unsupported and unanimous (k = 4) edges, each with its evidence sentence and audit note.

Supplementary Table 11 expanded gold standard (n = 151): round-1, round-2 and pooled expert precision per model, with Wilson 95% confidence intervals under both the strict and the lenient adjudication rule.

## References

[1] Pardoll DM . The blockade of immune checkpoints in cancer immunotherapy. Nat Rev Cancer. (2012) 12:252–64. doi: 10.1038/nrc3239 22437870 PMC4856023

[2] Leach DR Krummel MF Allison JP . Enhancement of antitumor immunity by CTLA-4 blockade. Sci (New York NY). (1996) 271:1734–6. doi: 10.1126/science.271.5256.1734 8596936

[3] Hodi FS O'Day SJ McDermott DF Weber RW Sosman JA Haanen JB . Improved survival with ipilimumab in patients with metastatic melanoma. N Engl J Med. (2010) 363:711–23. doi: 10.1056/nejmoa1003466 20525992 PMC3549297

[4] Topalian SL Hodi FS Brahmer JR Gettinger SN Smith DC McDermott DF . Safety, activity, and immune correlates of anti-PD-1 antibody in cancer. N Engl J Med. (2012) 366:2443–54. doi: 10.1056/nejmoa1200690 22658127 PMC3544539

[5] Sharma P Allison JP . The future of immune checkpoint therapy. Sci (New York NY). (2015) 348:56–61. doi: 10.1126/science.aaa8172 25838373

[6] Ribas A Wolchok JD . Cancer immunotherapy using checkpoint blockade. Sci (New York NY). (2018) 359:1350–5. doi: 10.1126/science.aar4060 29567705 PMC7391259

[7] Larkin J Chiarion-Sileni V Gonzalez R Grob JJ Cowey CL Lao CD . Combined nivolumab and ipilimumab or monotherapy in untreated melanoma. N Engl J Med. (2015) 373:23–34. doi: 10.1056/nejmoa1504030 26027431 PMC5698905

[8] Borghaei H Paz-Ares L Horn L Spigel DR Steins M Ready NE . Nivolumab versus docetaxel in advanced nonsquamous non-small-cell lung cancer. N Engl J Med. (2015) 373:1627–39. doi: 10.1016/j.annonc.2023.10.004 26412456 PMC5705936

[9] Reck M Rodríguez-Abreu D Robinson AG Hui R Csőszi T Fülöp A . Pembrolizumab versus chemotherapy for PD-L1-positive non-small-cell lung cancer. N Engl J Med. (2016) 375:1823–33. doi: 10.1056/nejmoa1606774 27718847

[10] Robert C Schachter J Long GV Arance A Grob JJ Mortier L . Pembrolizumab versus ipilimumab in advanced melanoma. N Engl J Med. (2015) 372:2521–32. doi: 10.1056/nejmoa1503093 25891173

[11] Tawbi HA SChadendorf D Lipson EJ Ascierto PA Matamala L Castillo Gutiérrez E . Relatlimab and nivolumab versus nivolumab in untreated advanced melanoma. N Engl J Med. (2022) 386:24–34. doi: 10.1056/nejmoa2109970 34986285 PMC9844513

[12] Rizvi NA Hellmann MD Snyder A Kvistborg P Makarov V Havel JJ . Cancer immunology. Mutational landscape determines sensitivity to PD-1 blockade in non-small cell lung cancer. Sci (New York NY). (2015) 348:124–8. doi: 10.1126/science.aaa1348 25765070 PMC4993154

[13] Sharma P Hu-Lieskovan S Wargo JA Ribas A . Primary, adaptive, and acquired resistance to cancer immunotherapy. Cell. (2017) 168:707–23. doi: 10.1016/j.cell.2017.01.017 28187290 PMC5391692

[14] Nicholson DN Greene CS . Constructing knowledge graphs and their biomedical applications. Comput Struct Biotechnol J. (2020) 18:1414–28. doi: 10.1016/j.csbj.2020.05.017 32637040 PMC7327409

[15] Himmelstein DS Lizee A Hessler C Brueggeman L Chen SL Hadley D . Systematic integration of biomedical knowledge prioritizes drugs for repurposing. eLife. (2017) 6. doi: 10.7554/elife.26726 28936969 PMC5640425

[16] Chandak P Huang K Zitnik M . Building a knowledge graph to enable precision medicine. Sci Data. (2023) 10:67. doi: 10.1038/s41597-023-01960-3 36732524 PMC9893183

[17] Zitnik M Agrawal M Leskovec J . Modeling polypharmacy side effects with graph convolutional networks. Bioinf (Oxford England). (2018) 34:i457–66. doi: 10.1093/bioinformatics/bty294 29949996 PMC6022705

[18] Percha B Altman RB . A global network of biomedical relationships derived from text. Bioinf (Oxford England). (2018) 34:2614–24. doi: 10.1093/bioinformatics/bty114 29490008 PMC6061699

[19] Swanson DR . Fish oil, Raynaud's syndrome, and undiscovered public knowledge. Perspect Biol Med. (1986) 30:7–18. doi: 10.1353/pbm.1986.0087 3797213

[20] Bordes A Usunier N Garcia-Duran A Weston J Yakhnenko O . “ Translating embeddings for modeling multi-relational data”, in: Advances in Neural Information Processing Systems (NeurIPS), Red Hook, NY: Curran Associates, Inc (2013). 26, 2787–95.

[21] Sun Z Deng Z-H Nie J-Y Tang J . “ RotatE: Knowledge graph embedding by relational rotation in complex space”, in: Proceedings of ICLR. OpenReview.net (2019).

[22] Trouillon T Welbl J Riedel S Gaussier E Bouchard G . “ Complex embeddings for simple link prediction”, in: Proceedings of the 33rd International Conference on Machine Learning, PMLR (2016). 48:2071–80.

[23] Wang Q Mao Z Wang B Guo L . Knowledge graph embedding: A survey of approaches and applications. IEEE Trans Knowl Data Eng. (2017) 29:2724–43. doi: 10.1109/tkde.2017.2754499 25079929

[24] Rossi A Barbosa D Firmani D Matinata A Merialdo P . Knowledge graph embedding for link prediction: A comparative analysis. ACM Trans Knowl Discov Data. (2021) 15:1–49. doi: 10.1145/3424672

[25] Barabasi A Albert R . Emergence of scaling in random networks. Sci (New York NY). (1999) 286:509–12. doi: 10.1126/science.286.5439.509 10521342

[26] Du S Yang B Wang X Li W-Y Lu X-H Zheng Z-H . Identification of potential leukocyte antigen-related protein (PTP-LAR) inhibitors through 3D QSAR pharmacophore-based virtual screening and molecular dynamics simulation. J Biomol Struct Dyn. (2020) 38:4232–45. doi: 10.1080/07391102.2019.1676825 31588870

[27] Yang B Li X He L Zhu Y . Computer-aided design of temozolomide derivatives based on alkylglycerone phosphate synthase structure with isothiocyanate and their pharmacokinetic/toxicity prediction and anti-tumor activity. Biomed Rep. (2018) 8:235–40. doi: 10.3892/br.2018.1051 29599977 PMC5867473

[28] Gao Y Li H Qin C Yang B Ke Y . Embryonic stem cells-derived exosomes enhance retrodifferentiation of retinal Muller cells by delivering BDNF protein to activate Wnt pathway. Immunobiology. (2022) 227:152211. doi: 10.1016/j.imbio.2022.152211 35390666

[29] Wang Q Wang Y Du L Xu C Sun Y Yang B . shRNA-mediated XRCC2 gene knockdown efficiently sensitizes colon tumor cells to X-ray irradiation *in vitro* and *in vivo*. Int J Mol Sci. (2014) 15:2157–71. doi: 10.3390/ijms15022157 24481064 PMC3958843

[30] Brown TB Mann B Ryder N Subbiah M Kaplan J . “ Language models are few-shot learners”, in: Advances in Neural Information Processing Systems (NeurIPS), Red Hook, NY: Curran Associates, Inc (2020). 33, 1877–901.

[31] OpenAI . GPT-4 technical report. arXiv preprint. (2023) arXiv:2303.08774. doi: 10.48550/arXiv.2303.08774

[32] Singhal K Azizi S Tu T Mahdavi SS Wei J Chung HW . Large language models encode clinical knowledge. Nature. (2023) 620:172–80. doi: 10.1038/s41586-023-06291-2 37438534 PMC10396962

[33] Agrawal M Hegselmann S Lang H Kim Y Sontag D . Large language models are few-shot clinical information extractors, in: Proceedings of EMNLP 2022, Abu Dhabi: Association for Computational Linguistics (2022). 1998–2022. doi: 10.18653/v1/2022.emnlp-main.130

[34] Wei X Cui X Cheng N Wang X Zhang X Huang S . ChatIE: Zero-shot information extraction via chatting with ChatGPT. arXiv preprint. (2023) arXiv:2302.10205. doi: 10.48550/arXiv.2302.10205

[35] Pan S Luo L Wang Y Chen C Wang J Wu X . Unifying large language models and knowledge graphs: A roadmap. IEEE Trans Knowl Data Eng. (2024) 36:3580–99. doi: 10.1109/tkde.2024.3352100 25079929

[36] Lee J Yoon W Kim S Kim D Kim S So CH . BioBERT: A pre-trained biomedical language representation model for biomedical text mining. Bioinf (Oxford England). (2020) 36:1234–40. doi: 10.1093/bioinformatics/btz682 31501885 PMC7703786

[37] Gu Y Tinn R Cheng H Lucas M Usuyama N Liu X . Domain-specific language model pretraining for biomedical natural language processing. ACM Trans Comput Healthcare. (2021) 3:1–23. doi: 10.1145/3458754

[38] Ji Z Lee N Frieske R Yu T Su D Xu Y . Survey of hallucination in natural language generation. ACM Comput Surv. (2023) 55:1–38. doi: 10.1145/3571730

[39] DeepSeek-AI . DeepSeek-V3 technical report. arXiv preprint. (2024) arXiv:2412.19437. doi: 10.48550/arXiv.2412.19437

[40] Bai J Bai S Chu Y Cui Z Dang K al e . Qwen technical report. arXiv preprint. (2023) arXiv:2309.16609. doi: 10.48550/arXiv.2309.16609

[41] Zeng A Liu X Du Z Wang Z Lai H . “ GLM-130B: An open bilingual pre-trained model”, in: Proceedings of ICLR. OpenReview.net (2023).

[42] Cohen J . A coefficient of agreement for nominal scales. Educ psychol Measurement. (1960) 20:37–46. doi: 10.1177/001316446002000104

[43] Feinstein AR Cicchetti DV . High agreement but low kappa: I. The problems of two paradoxes. J Clin Epidemiol. (1990) 43:543–9. doi: 10.1016/0895-4356(90)90158-l 2348207

[44] Byrt T Bishop J Carlin JB . Bias, prevalence and kappa. J Clin Epidemiol. (1993) 46:423–9. doi: 10.1016/0895-4356(93)90018-v 8501467

[45] Zheng L Chiang W-L Sheng Y Zhuang S Wu Z . “ Judging LLM-as-a-judge with MT-bench and chatbot arena”, in: Advances in Neural Information Processing Systems (NeurIPS Datasets and Benchmarks Track), Red Hook, NY: Curran Associates, Inc (2023). 36.

[46] Szklarczyk D Kirsch R Koutrouli M Nastou K Mehryary F Hachilif R . The STRING database in 2023: Protein-protein association networks and functional enrichment analyses for any sequenced genome of interest. Nucleic Acids Res. (2023) 51:D638–46. doi: 10.1093/nar/gkac1000 36370105 PMC9825434

[47] Kanehisa M Goto S . KEGG: Kyoto encyclopedia of genes and genomes. Nucleic Acids Res. (2000) 28:27–30. doi: 10.1093/nar/28.1.27 10592173 PMC102409

[48] Zarin DA Tse T Williams RJ Califf RM Ide NC . The ClinicalTrials.gov results database--update and key issues. N Engl J Med. (2011) 364:852–60. doi: 10.1056/nejmsa1012065 21366476 PMC3066456

[49] Baker M . 1,500 scientists lift the lid on reproducibility. Nature. (2016) 533:452–4. doi: 10.1038/533452a 27225100

[50] Dietterich TG . “ Ensemble methods in machine learning”, in: Multiple Classifier Systems (MCS 2000), Lecture Notes in Computer Science, Berlin, Heidelberg: Springer. (2000) 1857, 1–15. doi: 10.1007/3-540-45014-9_1

[51] Wang X Wei J Schuurmans D Le Q Chi E Narang S . “ Self-consistency improves chain-of-thought reasoning in language models”, in: Proceedings of ICLR. OpenReview.net (2023).

[52] Dogan RI Leaman R Lu Z . NCBI disease corpus: a resource for disease name recognition and concept normalization. J BioMed Inf. (2014) 47:1–10. doi: 10.1016/j.jbi.2013.12.006 PMC3951655 24393765

[53] Freshour SL Kiwala S Cotto KC Coffman AC McMichael JF Song JJ . Integration of the Drug-Gene Interaction Database (DGIdb 4.0) with open crowdsource efforts. Nucleic Acids Res. (2021) 49:D1144–51. doi: 10.1093/nar/gkaa1084 33237278 PMC7778926

[54] Weinstein JN Collisson EA Mills GB Shaw KRM Ozenberger BA Ellrott K . The Cancer Genome Atlas Pan-Cancer analysis project. Nat Genet. (2013) 45:1113–20. doi: 10.1038/ng.2764 24071849 PMC3919969

